# Assessing the burden and spatial distribution of *Taenia solium* human neurocysticercosis in Ecuador (2013–2017)

**DOI:** 10.1371/journal.pntd.0008384

**Published:** 2020-06-08

**Authors:** Marco Coral-Almeida, Aquiles R. Henriquez-Trujillo, Sofia Asanza, Celia Erazo, Michelle Paucar, Manuel Calvopiña

**Affiliations:** One Health Research Group, Faculty of Health Sciences, Universidad de Las Américas, Quito, Ecuador; IRNASA, CSIC, SPAIN

## Abstract

**Background:**

Estimating the burden of neglected tropical diseases is a valuable tool to support policymakers in the resource allocation for control and elimination of these diseases. Spatial analysis allows to identify the geographical distribution patterns of infectious and parasitic diseases within a country and allows to assess their possible correlation with other health disorders. Despite being neurocysticercosis (NCC) considered as the most important parasitic disease of the nervous system, few efforts have been addressed to assess the real burden of NCC in endemic countries, to date, there are no studies estimating the burden of NCC in South America. In this study we aimed to use the Disability Adjust Life Years (DALY) and spatial indicators as tools to measure the impact of human neurocysticercosis in Ecuador between 2013 and 2017.

**Methods:**

Mortality, morbidity and spatial data from the national agency of statistics were used to estimate the burden of disease of NCC during a five-year period (2013–2017). NCC cases and its two main sequelae, epilepsy and migraine headache, were stratified by sex and age group to calculate the DALY associated to NCC using the DALY package in R. SATSCAN software was used to assess spatial clusters of NCC and its possible neurological sequelae as epilepsy, status epilepticus, migraine and hydrocephalus.

**Principal findings:**

The burden of human neurocysticercosis ranged from 56201 [95% CI 29961–89333] to 59612 [95% CI 31854–94689] DALY per year, corresponding to 3.54 to 3.56 DALY per 1000 population. Average yearly incidence rates per 10 000 person-years were 0.23 [95% CI 0.21–0.26] for NCC, 4.89 [95% CI 4.78–5.00] for epilepsy, 0.130 [95% CI 0.11–0.15] for status epilepticus, 0.62 [95% CI 0.58–0.66] for migraine headache, and 1.02 [95% CI 0.98–1.07] for hydrocephalus. Most important significant spatial clusters (p<0.0001) were located in the southern region of the highlands of the country.

**Conclusion/Significance:**

This is the first study in South America to calculate estimates for burden of NCC and one of the few using spatial analysis to show the importance of sequelae other than epilepsy that play an important role in the impact of human neurocysticercosis.

## Author summary

*Taenia solium*-neurocysticercosis (NCC) is a neglected parasite infection in humans causing a variety of neurological sequelae like epilepsy, and chronic headache. The purpose of this study was to estimate the burden of NCC in Ecuador using official nation-wide databases from the National Institute of Statistics and Census (INEC) for the period of 2013–2017, and to assess for spatial clusters of four neurological disorders associated with NCC. The burden of NCC measured in Disability Adjusted Life Years (DALY) was estimated in 3.54 to 3.56 DALY per 1000 population. Several significant spatial clusters for augmented risk of disease were identified along the country for NCC and its sequelae, most of which converged in the southern Sierra provinces. Our study suggests a possible spatial correlation between the presence of NCC cases and a higher prevalence of neurological conditions like epilepsy, status epilepticus, migraine, and hydrocephalus in several ‘hot spots’ of the southern provinces of Ecuador, indicating possible areas where the application of preventive measures is necessary to reduce cysticercosis transmission rates.

## Introduction

Human neurocysticercosis (NCC) is a neglected tropical zoonotic parasitic disease caused by the larval stage of the cestode *Taenia solium* tapeworm. The natural cycle of the disease includes pigs as intermediate hosts and humans as definitive hosts. Pigs acquire the *T*. *solium* metacestode (cysticercus) after ingestion of eggs shed in human feces of tapeworm carriers, the larval stage of *T*. *solium* then stablishes in muscles and other inner tissues of the pig and develops in a viable cysticercus [1]. Humans are the sole definitive host, developing the adult *T*. *solium* tapeworm in their intestines after ingestion of pork with viable cysticerci. Once the parasite is fully developed in an adult, the human host can shed thousands of eggs to the environment through defecation [2]. Human cysticercosis occurs when humans become accidentally infected with *T*. *solium* eggs through oral ingestion of food or water contaminated with human feces of tapeworm carriers, then the metacestode establishes in the human host inner tissue [3]. When the metacestode is located in the Central Nervous System (CNS) the disease is called neurocysticercosis (NCC) [4]. The life cycle of the parasite is maintained by poor hygienic conditions, poverty, open defecation and free roaming pigs, these conditions are often found in endemic regions for *T*. *solium* in Africa, Asia and Latin-America [5]. NCC can cause different neurological disorders, going from asymptomatic and mild disorders, chronic primary headache (mainly migraine), to severe types of nervous disorders, such as epilepsy, status epilepticus, hydrocephalus and death [6–12].

Limited resources countries are often endemic for *T*. *solium*. The correct diagnosis of NCC requires the use of multiple tools that are not always available to all patients in limited resources countries, thus, many neurological disorders cannot be properly identified and remain reported as idiopathic [4,13–15]. To these days, the causal relationship between NCC and different neurological disorders, as well as the specific proportion of epilepsy and other neurological disorders in the tropics directly associated with NCC cases, remain uncertain [16,17], however, evidence still shows that NCC is the most important parasitic disease of the CNS and plays a significant role for epilepsy cases in endemic countries [18]. In these conditions, estimating the real impact of neglected diseases such as NCC remains a challenge, therefore, complicating decision-making [19–22].

Ecuador is a Latin American Andean country hyperendemic for NCC [23]. Sero-epidemiological studies conducted in Ecuador reported active infections in humans with prevalences for circulating antigens of *T*. *solium* varying from 0.94% [95% CI 0.38–1.93] to 4.99% [95% CI 4.36–5.69], while exposure to the parasite has been reported with seroprevalences of antibodies directed to *T*. *solium* cysticerci varying from 25.3% [95% CI 22.08–28.17] to 40% [95% CI 30.33–50.23] [24–27].

Previous studies in Ecuador have shown the heterogeneity of the geographical distribution of NCC cases within the country, which should be taken into account in order to avoid over/underestimation of the real impact of NCC, as well as, for other neglected tropical diseases. Spatial analysis of the distribution of NCC cases and other potentially associated neurological disorders could bring more evidence to the discussion of the role of NCC in the occurrence of CNS disorders in *T*. *solium* endemic regions [28].

The disability-adjusted life year (DALY) metrics is an indicator of disease burden which combines the years lived with disability (YLD) and the years of life lost due to premature death (YLL). The DALY metric has been widely used by the Global Burden of Disease (GBD) study to help policymakers in their decisions, quantifying the non-economic impact of a disease in a country or region [29]. Many authors call the urgency of reporting the impact of NCC in different endemic regions in order to help to improve the international comparison of disease burden and to identify priorities in decision-making [19,30,31], however, global- and national-level estimates can mask local variations within national borders and, to date, there is a lack of studies estimating the burden of NCC in South America. This article aims to estimate the burden of NCC at the national and subnational level using available national databases for the period of 2013–2017, and assess for possible spatial correlation between the presence of NCC cases and a higher prevalence of neurological conditions like epilepsy, status epilepticus, migraine, and hydrocephalus at the subnational level.

## Materials and methods

### Ethics statement

This project was approved by the Universidad de Las Américas Institutional Review Board, which granted a waiver for IRB review. Data were obtained from publicly available databases from the Ecuadorian National Institute of Statistics and Census (INEC) and the Ecuadorian Ministry of Public Health (MoH) and by legal mandate all records are deidentified [32].

### Geographical location and study population

Ecuador is located in the Pacific coast of South America, limiting to the north with Colombia and to the South and East with Peru. Ecuador has four geographical regions: the Galapagos Islands, the coastal region, the highlands of the Andean mountains, and the Amazonia. There are little seasonal variations in the temperature the whole year, whereas there are significant variations between regions. Galapagos Islands have a dry and warm weather; coastal and Amazonia regions possess similar landscapes with tropical rainforests; and the Andean highlands have temperate temperatures. The country is divided in 24 provinces, and 224 cantons. The last nation-wide census in year 2010 recorded 15,012,228 inhabitants, while the INEC estimated for 2019 the population of Ecuador to be 17,267,986 [33].

### Sources of information and study design

This is a cross-sectional retrospective study. Registries of all deaths and hospital discharges reported at the national level by the General Direction of Civil Registry and the Ecuadorian Ministry of Health for the period 2013–2017 were downloaded in.csv format from the National Archive of Data and Statistical Metadata of the Ecuadorian National Institute of Statistics and Censuses (INEC) available at https://anda.inec.gob.ec/anda/index.php/catalog. ICD-10 code was used to identify deaths and hospital admissions due to cysticercosis of central nervous system(B69.0) and four neurological disorders potentially associated with neurocysticercosis (NDPAN): epilepsy (G40), status epilepticus (G41), migraine (G43), and hydrocephalus (G91) [34,35]. Data were preprocessed in Stata v16 before its analysis in R v3.6.1.

### Estimation of the burden of disease

The burden of disease attributable to NCC during the study period was measured using DALY, the sum of years lived with disability (YLDs) and years of life lost due to premature mortality (YLLs), following the methods described by Murray et al. for the GBD studies [36–39]. Calculations were made using the “DALY” package for R [40].

YLLs were estimated as the product of the number of deaths registered due to NCC in the study period, and the residual life expectancy at the age of death. To estimate residual life expectancy, the Coale and Demeny model life table West 26 was used with a life expectancy at birth of 80 years for males and 82.5 years for females [36]. For DALY calculations we used a time discount rate of 3% per year to reflect the preference on life years closer to the present, but without age weighting to avoid lower weights to years of healthy life at very young and old ages [36,41].

Available data from consultations and hospital admissions registered by the public healthcare services are only a subset of the symptomatic NCC population with effective access to healthcare and not suitable for YLD estimations at the country level. Therefore to identify some of the parameters for YLD calculations we performed a literature search in PubMed using a combination of the terms “neurocysticercosis[mh] AND burden[ti]”, “neurocysticercosis[mh] AND Ecuador[mh]”, and “neurocysticercosis[mh] AND Latin America[mh]” and a snowball method to find other relevant titles from the references. A prevalence-based approach was used to calculate the number of expected cases of NCC at the population level [30]. Estimation of YLD due to NCC were calculated using the pooled estimates of the proportion of clinical manifestations among symptomatic NCC patients published by Carabin et al. in year 2011[11]. Epilepsy and migraine headache were considered as the two main sequela of NCC. Disability weights (DW) from the estimates by the Global Burden of Disease 2017 study for epilepsy 0.263 [95% CI 0.173–0.367] and migraine 0.441 [95% CI 0.294–0.588] were used for YLD calculations [42]. Based on the prevalence of epilepsy and migraine, the number of people with neurocysticercosis-associated sequela was estimated. The reported point prevalence of active epilepsy in Ecuador is 7 to 12 cases per 1000 population, with an incidence rate between 120 to 172 new cases per 100 000 person-years [43]. For the Ecuadorian population the probability of seizures recurrence in patients with epilepsy is estimated in 30% at 12 months [43].

Prevalence of migraine was obtained from the case control study by Del Brutto et al. reporting a significant association between migraine headache and calcified NCC (OR 4.89 [95% CI 2.36–11.39]). In this population 62.2% and 43.3% of patients with NCC reported current or intense headaches, respectively [44]. The estimated prevalence of calcified NCC among patients with primary headache in Ecuadorian population is 4.72% [95% CI 3.47% - 6.26%] [45]. Table 1 describes the parameters used for burden estimations and their probability distributions used in the DALY package for R.

**Table 1:** Parameters used for the burden estimation of NCC in the DALY calculator for R.

Parameter	Probability distribution	Value range	Source
Population	Fixed by age and sex	15.77 million in year 2013 to 16.77 million in year 2017	INEC 2019 [33]
Prevalence of epilepsy (G40)	Uniform	0.007 to 0.012	Carpio et al. 2001 [43]
Prevalence of epilepsy associated to NCC	Uniform	0.00057 to 0.00276	Carpio et al. 2001[43]; Del Brutto et al. 2018 [44]
Proportion of recurrent seizures at 1 year	Fixed	0.3	Carpio et al. 2001 [43]
Prevalence of migraine headache (G43)	Uniform	0.127 to 0.144	Del Brutto et al. 2018 [44]
Prevalence of migraine headache associated to NCC	Uniform	0.047 to 0.057	Del Brutto et al. 2012 [45]
Disability weight for idiopathic, seizure-free, treated epilepsy.	Uniform	0.031 to 0.072	Global Burden of Disease study [42]
Disability weight for epilepsy, seizures 1–11 per year.	Uniform	0.173 to 0.367	Global Burden of Disease study [42]
Disability weight for idiopathic, seizure-free, treated epilepsy.	Uniform	0.233 to 0.537	Global Burden of Disease study [42]
Disability weight for migraine headache	Uniform	0.294 to 0.588	Global Burden of Disease study [42]
Average duration of disability in years in males by age group			Praet et al. [30]
0–4 years	Fixed	1.4	
5–14 years	Fixed	2	
15–44 years	Fixed	3.6	
45–59 years	Fixed	2.8	
60+ years	Fixed	1.6	
Average duration of disability in years in females by age group			Praet et al. [30]
0–4 years	Fixed	1.6	
5–14 years	Fixed	3.1	
15–44 years	Fixed	5.9	
45–59 years	Fixed	6	
60+ years	Fixed	2.8	

### Spatial analysis and statistical methods

The spatial analysis was conducted in order to identify the important spatial clusters for the cantons with significant higher incidence rates of hospitalized cases of: 1) NCC, 2) epilepsy, 3) status epilepticus, 4) migraine and 5) hydrocephalus [46,47]. Each NCC and NDPAN case was distributed geographically by canton and by ICD-10 identification code in order to obtain the statistically significant spatial clusters for each diagnosis. Spatial data were only available in the official records for the 2013–2015 period.

The spatial analysis was conducted in SATSCAN v9.6 (Last version March 2018)[48], it searched, tested for significance and identified approximate locations of areas with an increased incidence rate for the occurrence of NCC and four NDPAN, following the methodology described by Ron-Garrido et al.[28] and Kulldorff [46] with small modifications for purely spatial analysis. Briefly, the purely spatial analysis used the number of reported cases for each NDPAN distributed by canton together with the total population of the canton and the spatial coordinates of each canton, then, a Poisson distribution was used to compare the number of cases in the scanned locations. Space clustering was assessed by comparing the incidence rate ratio (iRR) of the cases of NCC and the cases of NDPAN within a specific area in contrast to an expected iRR of the cases of NCC and the cases of NDPAN if their incidences were randomly distributed. The likelihood ratio test was used to check the significance of identified space clusters; p-values of the test were obtained using 999 Monte Carlo simulations. A cluster was identified as significant when obtained p-values were inferior to 0.05 [28]. An additional selection amongst the significant clusters was made using the Gini coefficient as described by Han et al. [49]. Visualization of the spatial analysis was done using QGIS version 3.8 Zanzibar software [50]. All maps were created and designed by the authors of this manuscript. Shape files for all the maps in this article were obtained from the INEC portal[51] following their licensing requirements (https://www.ecuadorencifras.gob.ec/registro-de-descargas-cartograficas/).

Incidence rates were standardized using the population projections for each year of the period of study adjusted by sex and age (details in supplementary information S3 and S4 Files). Incidences are reported in absolute numbers of new cases and relative rates per 10,000 inhabitants. Exact 95% Poisson confidence intervals (95% CI) were used for the report incidence rates and were calculated using the epitools package in R (version 3.6.0) software[40].

The spatial and numerical data used for all maps are included in S5 File.

## Results

During the period of 2013–2017, 1874 cases of hospitalized NCC, 39772 hospitalized cases of epilepsy, 1062 hospitalized cases of status epilepticus, 5047 hospitalized cases of migraine and 8335 hospitalized cases of hydrocephalus were reported in Ecuador. The corresponding yearly mean incidence rates were: 0.230 cases of NCC per 10 000 person-years [95% CI 0.208–0.255], 4.887 cases of epilepsy per 10 000 population [95% CI 4.780–4.995], 0.130 cases of status epilepticus per 10 000 person-years [95% CI 0.113–0.149], 0.620 cases of migraine per 10 000 person-years [95% CI 0.582–0.659] and 1.024 cases of hydrocephalus per 10 000 person-years [95%CI 0.976–1.074]. Specifically, for NCC hospitalized cases, Table 2 describes the distribution by sex, age group and by area of residency.

**Table 2:** Distribution by sex, age group and area of residency of hospitalizations due to neurocysticercosis in Ecuador, 2013–2017.

	Years of study (number of neurocysticercosis cases)									
	2013	(n = 266)	2014	(n = 205)	2015	(n = 199)	2016	(n = 162)	2017	(n = 175)
Variable	n	%	n	%	n	%	n	%	n	%
**Sex**										
male	134	50.40	119	58.00	101	50.80	85	52.50	102	58.29
female	132	49.60	86	42.00	98	49.20	77	47.50	73	41.71
**Age group**										
0	1	0.38	---	---	---	---	---	---	---	---
1–4	7	2.63	5	2.44	---	---	1	0.62	---	---
5–9	7	2.63	6	2.93	5	2.51	2	1.23	5	2.86
10–14	12	4.51	12	5.85	4	2.01	7	4.32	2	1.14
15–19	7	2.63	7	3.41	5	2.51	11	6.79	9	5.14
20–24	15	5.64	9	4.39	4	2.01	12	7.41	13	7.43
25–29	15	5.64	12	5.85	9	4.52	20	12.35	14	8
30–34	28	10.53	15	7.32	21	10.55	8	4.94	29	16.57
35–39	20	7.52	14	6.83	13	6.53	12	7.41	16	9.14
40–44	21	7.89	17	8.29	21	10.55	12	7.41	13	7.43
45–49	25	9.4	13	6.34	27	13.57	11	6.79	5	2.86
50–54	16	6.02	13	6.34	14	7.04	14	8.64	12	6.86
55–59	18	6.77	20	9.76	15	7.54	10	6.17	8	4.57
60–64	17	6.39	15	7.32	13	6.53	8	4.94	14	8
65–69	12	4.51	15	7.32	11	5.53	7	4.32	12	6.86
70–74	15	5.64	18	8.78	13	6.53	6	3.7	8	4.57
75–79	14	5.26	5	2.44	13	6.53	8	4.94	3	1.71
80–84	9	3.38	3	1.46	2	1.01	9	5.56	3	1.71
85+	7	2.63	6	2.93	9	4.52	4	2.47	9	5.14
Area of residency*										
Urban					168	84.40	140	86.40	147	84.00
Rural					31	15.60	22	13.60	28	16.00

*Data available only for years 2015 to 2017.

### Burden of disease

The yearly burden of disease ranged from 56201 to 59612 DALY per year, which corresponded to 3.54 to 3.56 DALY per 1000 population. The number of registered deaths associated with NCC, the annual number of estimated incident cases of NCC, the YLLs and YLDs associated with epilepsy and headache due to NCC, the estimated annual DALYs lost and the annual DALY rate are described in Table 3.

**Table 3:** Burden of disease estimates due to human neurocysticercosis in Ecuador between years 2013–2017.

Burden estimates	2013	2014	2015	2016	2017
Deaths registered—n	21	12	10	13	8
Estimated cases—n (95% CI)	130390 (93221 to 166867)	132390 (95123 to 169649)	134425 (96255 to 172361)	136429 (97608 to 174826)	138374 (99660 to 177451)
Calculated total YLL—n	509	298	247	321	197
Estimated total YLD—n (95% CI)	55692 (29452 to 88824)	56702 (30180 to 90228)	57448 (30601 to 91631)	58420 (30939 to 93115)	59416 (31657 to 94492)
due to epilepsy	10559 (3697 to 19483)	10732 (4056 to 20047)	10910 (3838 to 20179)	11088 (3857 to 20369)	11280 (3913 to 20795)
due to headache	45133 (25755 to 69341)	45970 (26422 to 70479)	46538 (26763 to 71452)	47332 (27082 to 72746)	48136 (27744 to 73697)
Estimated total DALY—n (95% CI)	56201 (29961 to 89333)	57000 (30478 to 90526)	57695 (30848 to 91878)	58740 (31260 to 93435)	59612 (31854 to 94689)
DALY rate* - n (95% CI)	3.56 (1.89 to 5.66)	3.56 (1.90 to 5.65)	3.54 (1.89 to 5.64)	3.55 (1.89 to 5.65)	3.55 (1.90 to 5.64)

*Rate per 1000 population.

### Spatial analysis

**Fig 1:**
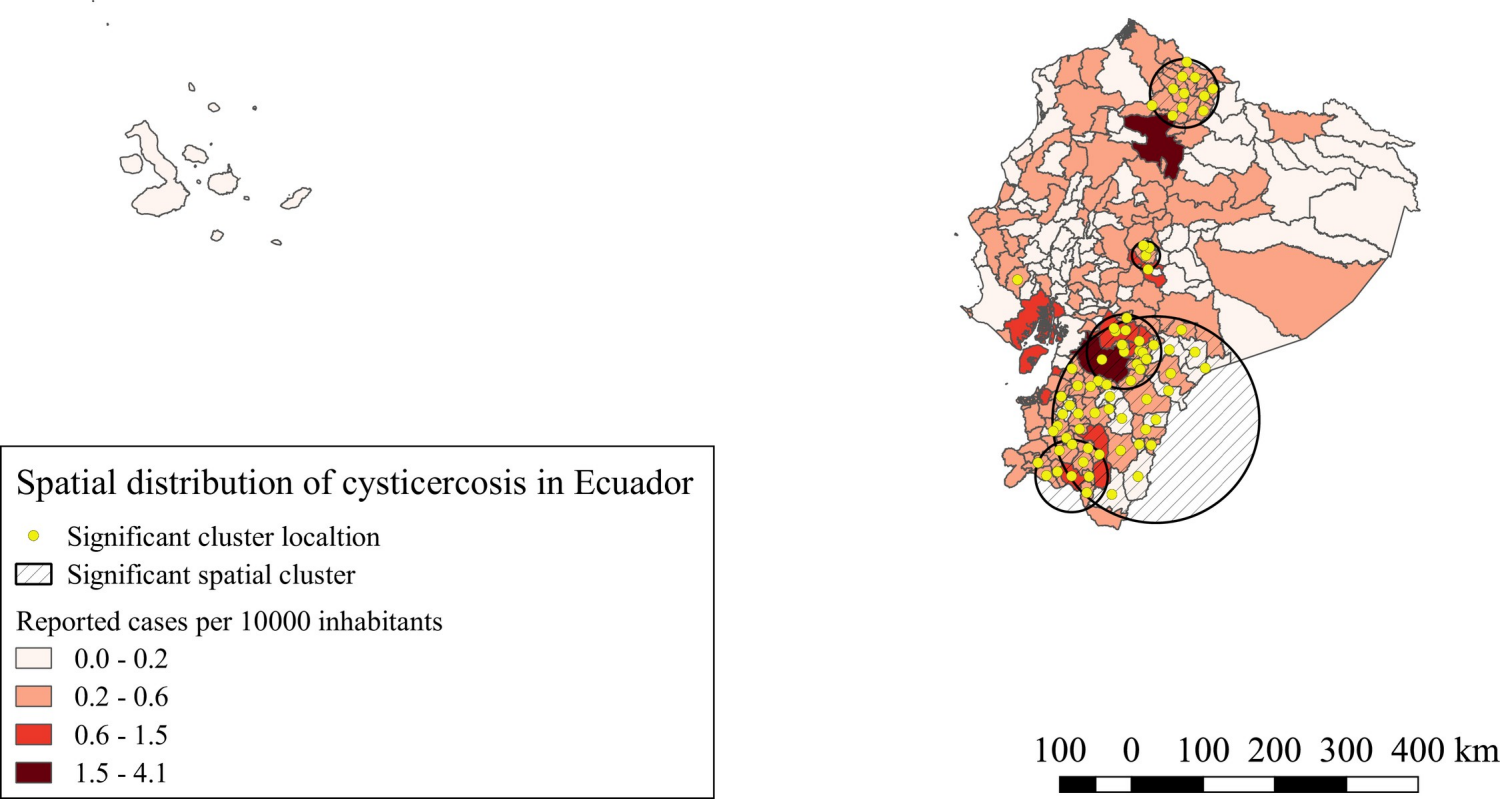
Spatial distribution and significant space clusters of NCC hospitalized cases in Ecuador. Shape files for this map were obtained from INEC portal [51].

For NCC hospitalized cases, seven significant spatial clusters were identified (p<0.0001). The iRR of NCC of these clusters varied from 2.10 to 5.15 compared to the areas outside the clusters. These clusters are located mostly in the northern and southern provinces of the highlands of Ecuador together with one south eastern province of the Amazonia. Other small clusters were located on the center of the highlands and a single canton cluster located in the coast, as is shown in Fig 1.

**Fig 2:**
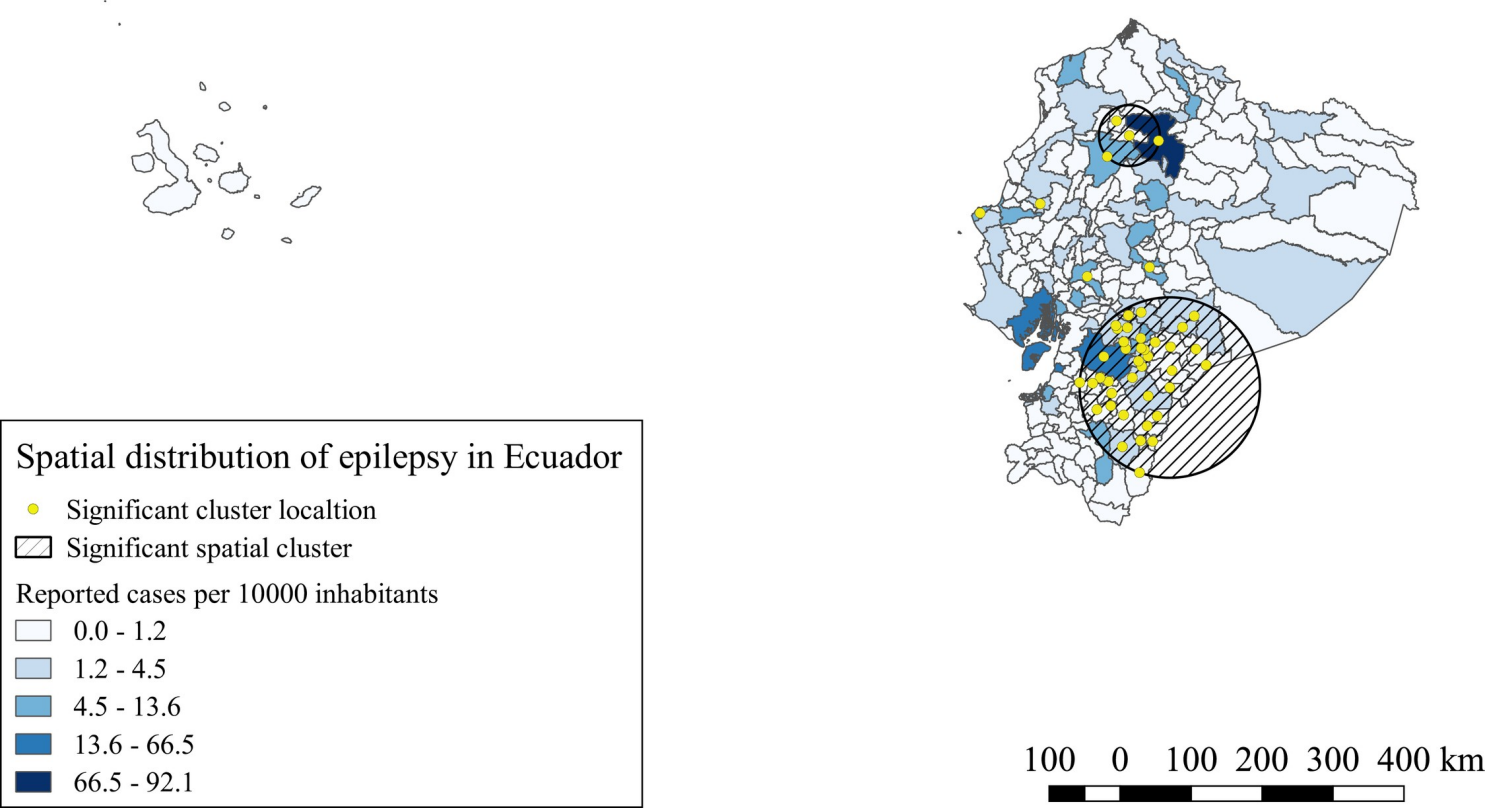
Spatial distribution and significant space clusters of epilepsy hospitalized cases in Ecuador. Shape files for this map were obtained from INEC portal [51].

For hospitalized epilepsy cases, six significant space clusters were identified (p<0.0001). The iRR of these clusters varied from 1.27 to 2.06 compared to the areas outside the clusters. The most representative clusters were located in the south and in the center north of the highlands, with two single canton clusters dispersed in the center of the highlands and other two single canton clusters in the center of the coast, as described in Fig 2.

**Fig 3:**
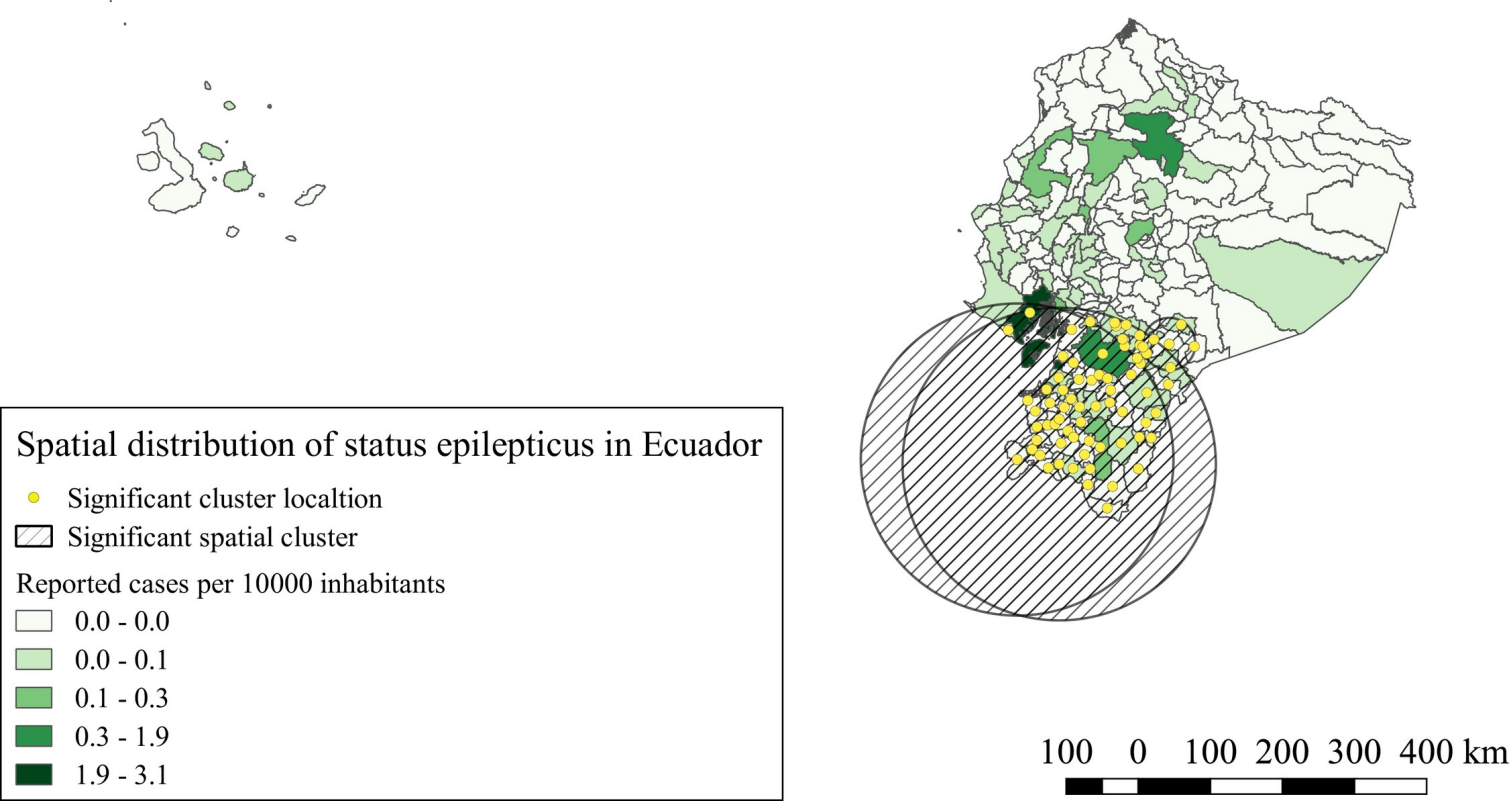
Spatial distribution and significant space clusters of status epilepticus hospitalized cases in Ecuador. Shape files for this map were obtained from INEC portal [51].

For status epilepticus hospitalized cases, three significant clusters were identified (p<0.0001). The iRR of these clusters varied from 2.06 and 8.54 compared to the areas outside the clusters. All three clusters were located in the southern region of the country in provinces from the coast, highlands and Amazonia, as described in Fig 3.

**Fig 4:**
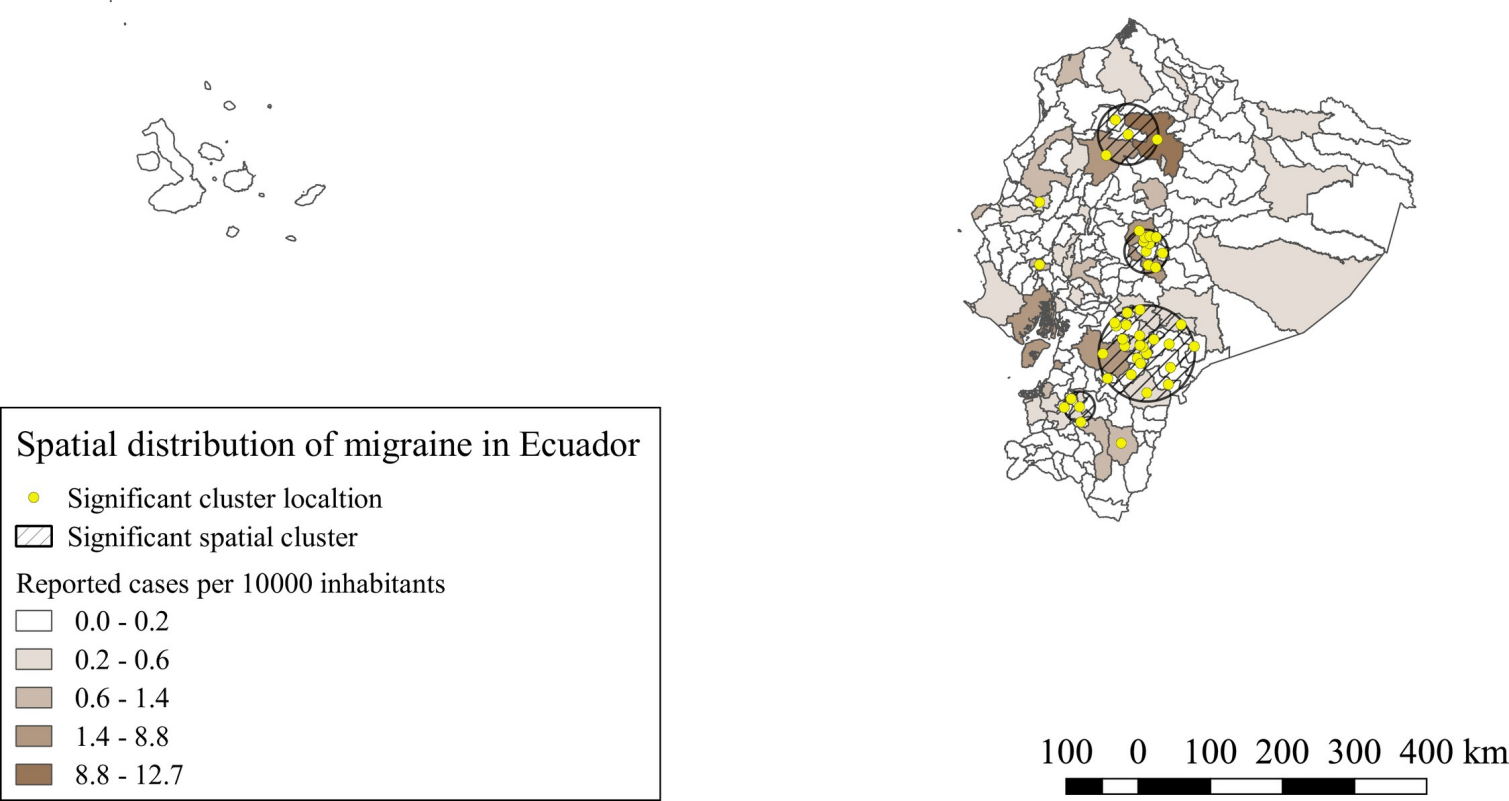
Spatial distribution and significant space clusters of migraine hospitalized cases in Ecuador. Shape files for this map were obtained from INEC portal [51].

For migraine hospitalized cases, seven significant spatial clusters were identified (p<0.0001). iRR of these clusters varied from 1.36 to 9.38 compared to the areas outside the clusters. The majority of the clusters were located in the provinces of the highlands, the four biggest clusters were located in the central-north, central and central south provinces of the highlands, three single canton clusters were also identified, two were identified in the central coastal provinces, whereas one was located in the a southern province of the highlands, as shown in Fig 4.

**Fig 5:**
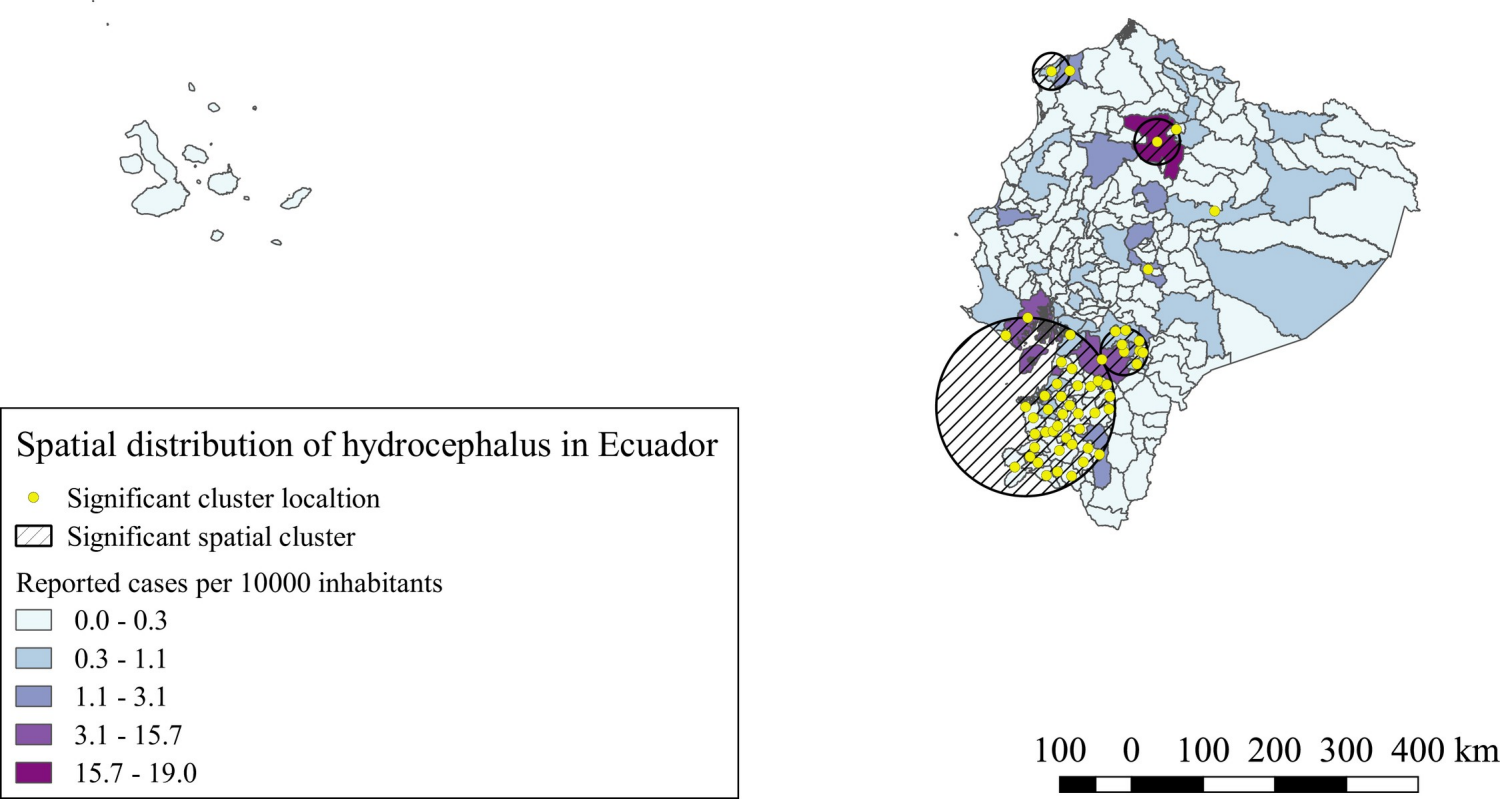
Spatial distribution and significant space clusters of hydrocephalus hospitalized cases in Ecuador. Shape files for this map were obtained from INEC portal [51].

For hospitalized hydrocephalus cases, 6 significant spatial clusters were identified (p<0.0001). IRR of these clusters varied from 1.15 to 3.03 compared to the areas outside the clusters. There were four bigger clusters, two in the southern part of the highlands and part of southern coast provinces, one more was located in the central-north provinces of the highlands and one in a northern province of coast, two single canton significant clusters were also identified in a central province of the highlands and one in the central-western part of the Amazonia, as shown in Fig 5.

## Discussion

This is the first study estimating the burden of disease of NCC in Ecuador and the first to analyze the spatial distribution of hospitalized cases of neurological conditions potentially associated with NCC, other than epilepsy. The number of hospitalized cases for NCC and epilepsy found in this study are consistent with the time trends found by Ron-Garrido et al. for the period of 1996–2008 in Ecuador [28]. The number of hospitalized cases of NCC reported in the present study for Ecuador were of 1874 cases in five years (2013–2017), which accounts for an average of 374.8 cases per year, these figures are lower than those previously reported by Ron-Garrido et al. in 2015, for their study period (1996–2008) they found 6294 cases, which accounts for 524.5 cases per year. However, this decrease in the number of hospitalized NCC cases is consistent the time trend also identified by the same authors which could be mainly due to the improvement in hygienic conditions. For epilepsy, 39772 cases were reported between 2013–2017 in this study (7954.4 cases per year), in the study by Ron-Garrido et al. the number of cases for 1996–2008 is much lower with 19821 cases reported (1651.75 cases per year), also consistent with the increasing trend described by the same authors that may be associated by an improvement in health facilities and coverage of hospitalization [28].

There are few studies estimating the burden of disease for NCC using DALYs. Four studies reporting DALYs for NCC are from Nepal, Laos, India and Cameroon [30,52–54]. For Latin America there is only one study published from Mexico[31]. Thus, the present article would be the first of its kind for South America. In our study, 57849.6 DALYs per year (3.552 per 1000 population) in average are attributable to NCC, 99% of these are attributable to disability (YLD) whereas only 1% is attributable to premature deaths, these figures are almost three times higher than those from the Mexican and Indian study, six times the Nepalese study, but almost one third of those reported by the study from Cameroon. These regional variations could be the result of many factors such as socio-economic, cultural, environmental and intrinsic factors affect the epidemiology of NCC resulting in the differences observed in the burden estimation [5], but these factors should be further explored in order to quantify the real impact of each to the epidemiology of *T*. *solium*, which should be translated in estimates that might be used to feed simulation models that can be used to assess the disease burden at regional and global level [24].

The GBD study 2016 estimated a total burden of 38919 DALYs for Ecuador attributable to epilepsy only [55], in our study, the number of DALYs attributable to NCC (including the corresponding proportion of epilepsy) was almost thirty percent higher, this disagreement between GBD estimates and country level studies was also noticed by Bhattarai et al. for their study in Mexico and by Praet et al. in their study in Cameroon [30,31]. Epilepsy burden should be higher than NCC burden if we take into account that NCC accounts only for a fraction of acquired epilepsies [16]. This disagreement could be the result of the variability in the parameters obtained from local studies and global projections, which increases the uncertainty around burden estimates. Regional projections can mask local variations within a country. Spatial analysis in our study shows clusters of NCC and NDPAN, which demonstrates heterogeneity in the distribution of NCC and NDPAN within the borders of Ecuador, for this reason, in our study, the parameters obtained from local studies are more accurate and conservative to estimate the burden of NCC in Ecuador than the parameters obtained from regional projections used in the GBD study, also, our burden estimations include epilepsy and headache as sequelae of NCC while the GBD study was using epilepsy as the only sequela, which results in more robust estimations from our study than those obtained by the GBD study.

The spatial analysis of our study, showed for NCC and the four NDPAN similar significant clusters, the southern provinces of the highlands, Amazonia and coast, bore the highest iRR and the majority of the significant clusters for all conditions. Similar results were observed by Ron-Garrido et al. for epilepsy and NCC [28], despite the different time period and the addition of new NDPAN, the spatial clusters are maintained, which indicates a potential strong correlation with these NDPAN and NCC, even though, not all epilepsies and NDPAN are the result of NCC infections [13,56,57], thus, the correct proportions attributable to NCC must still be defined. Further studies are needed to understand this relationship.

Our study presents some limitations. The use of hospital data does not register asymptomatic NCC cases or patients with mild symptoms, also, areas with poor health services will not be able to diagnose NCC, thus, failing to register NCC cases on those zones which results in an underestimation of the number of NCC cases and the loss of the place of origin of these, affecting the overall spatial analysis. Other limitations were the possibility of having duplicate data as official records do not allow to identify if one patient was admitted more than one time per year for the same cause. For the NDPAN and epilepsy, official hospital records do not conclude if the etiology of these disorders is directly associated to a *T*. *solium* larval infection or other etiologies. Similar to the study in Mexico [31], one of the main limitations in this study was the lack of knowledge in the frequency and disability weights of other NDPAN, without those parameters, burden estimation of NCC including other important NDPAN such as hydrocephalus cannot be calculated, which results in an underestimation of the burden of NCC. More population-based studies are needed to quantify the relationship, frequency and disability weights attributable to NCC and other NDPAN. Other limitation is the possibility that the parameters obtained from local studies from Ecuador to estimate the burden of NCC do not cover all the areas where NCC is present in the country, however, these parameters remain more accurate for the burden estimation in Ecuador than those obtained from regional projections used for the GBD study.

Despite these limitations, the results obtained in this study are reliable as the methodology used has been standardized and the data analyzed is representative of the whole country, the unit of study was the canton, thus the precision level of the analyses is high.

The results of this study can help to adjust the global estimations of the burden of disease for NCC, previous studies called the need for more studies of this nature in order to fill the gaps in methodology and estimates for NCC. Obtaining fine-tuned proportion estimates for all the important sequelae for NCC could help to fine tune burden estimations of NCC. The presence of *T*. *solium* and NCC are indicators of lack of hygiene and poverty, these spatial results can also be indicators of poor wealth distribution in a country and thus indicators of vulnerable zones. Other burden indicators, such as zDALYs, should be used to complement societal and economic burden estimations of zoonotic diseases in identified spatial clusters, in order to call to the attention of policy-makers for better resource allocation [52,58,59].

In conclusion, the present study gave for the first time burden of disease estimates for NCC in South America, which would feed the global knowledge about NCC real impact and would help to fine tune the current methods used in NCC burden of disease estimation. Also, this study raised the importance of NCC sequelae, other than epilepsy, that should immediately be taken into account to notice the importance of this neglected disease in developing countries.

## Supporting information

S1 File STROBE checklist. (DOCX)

S2 File Ethical clearance. (PDF)

S3 File Supplementary tables. (DOCX)

S4 File Projected population by age group between years 2010–2020 for Ecuador. (XLSX)

S5 File Supplementary data for spatial analysis. (XLSX)
