# Supplementary material for: Assessing the burden and spatial distribution of Taenia solium human neurocysticercosis in Ecuador (2013–2017)
Source: PLoS Negl Trop Dis. 2020 Jun 8;14(6):e0008384. doi: 10.1371/journal.pntd.0008384 (PMC7302800; doi:10.1371/journal.pntd.0008384)
Supplement: S2 File — (PDF) [file pntd.0008384.s002.pdf]

Quito, D. M. Ecuador

17 de enero de 2020

Marco Coral Almeida
Investigador

Presente

De mis consideraciones:

Por medio de la presente el Comité de Ética de Investigación en Seres Humanos de la Universidad de Las Américas **(CEISH-UDLA)** le informa que el Presidente del Comité procedió a revisar la lista de verificación de ausencia de riesgo del proyecto **"Assessing the burden and spatial distribution of Taenia solium human cysticercosis and its neurological sequelae in Ecuador (2013-2017)"**, donde se constata que el proyecto no presenta riesgo mínimo; no hay ningún tipo de muestra humana y por lo tanto el protocolo de investigación no requiere revisión por parte del Comité.

Al no existir riesgo mínimo y con base en la normativa interna de la Universidad, le informamos que no es necesaria la aprobación del Comité.

Atentamente,

Diego R. Chauvin P.
Presidente del Comité
Universidad de Las Américas
ceish@udla.edu.ec

COMITÉ DE ÉTICA Y BIOÉTICA
