# Supplementary material for: Assessing the burden and spatial distribution of Taenia solium human neurocysticercosis in Ecuador (2013–2017)
Source: PLoS Negl Trop Dis. 2020 Jun 8;14(6):e0008384. doi: 10.1371/journal.pntd.0008384 (PMC7302800; doi:10.1371/journal.pntd.0008384)
Supplement: S3 File — (DOCX) [file pntd.0008384.s003.docx]

**Supplementary information**

Age standardized hospitalization rates for NCC and neurological conditions probably related to NCC in Ecuador between years 2013-2017. All rates are calculated over 100 thousand population. Standard population for adjustment is the Ecuador 2010 census population. Calculations were made in Epidat v4.2.

| **Table s1. Hospitalization rates for cysticercosis of central nervous system (ICD-10: B69.0)** | | | | | |
| --- | --- | --- | --- | --- | --- |
| **Year** | **Cases** | **Crude** | **ASR** | **95% Confidence Interval** | |
| 2013 | 266 | 16.86 | 16.64 | 14.62 | 18.97 |
| 2014 | 205 | 12.79 | 12.47 | 10.97 | 14.38 |
| 2015 | 199 | 12.22 | 11.80 | 10.39 | 13.88 |
| 2016 | 162 | 9.80 | 9.55 | 7.78 | 11.41 |
| 2017 | 176 | 10.49 | 10.10 | 8.20 | 11.89 |
| Rates per 100 thousand person-year; ASR = Age standardized rate | | | | | |

| **Table s2. Hospitalization rates for Epilepsy (ICD-10: G40)** | | | | | |
| --- | --- | --- | --- | --- | --- |
| **Year** | **Cases** | **Crude** | **ASR** | **95% Confidence Interval** | |
| 2013 | 3518 | 223.01 | 225.61 | 218.39 | 233.13 |
| 2014 | 3989 | 248.89 | 252.61 | 244.61 | 260.58 |
| 2015 | 4231 | 259.91 | 265.65 | 257.76 | 266.62 |
| 2016 | 4438 | 268.50 | 276.19 | 267.99 | 284.63 |
| 2017 | 4782 | 285.03 | 294.93 | 286.68 | 296.65 |
| Rates per 100 thousand person-year; ASR = Age standardized rate | | | | | |

| **Table s3. Hospitalization rates for status epilepticus (ICD-10: G41)** | | | | | |
| --- | --- | --- | --- | --- | --- |
| **Year** | **Cases** | **Crude** | **ASR** | **95% Confidence Interval** | |
| 2013 | 138 | 8.75 | 8.86 | 7.27 | 10.41 |
| 2014 | 170 | 10.61 | 10.89 | 8.79 | 12.89 |
| 2015 | 99 | 6.08 | 6.10 | 4.70 | 7.44 |
| 2016 | 116 | 7.02 | 7.21 | 6.03 | 8.67 |
| 2017 | 133 | 7.93 | 8.14 | 6.73 | 9.91 |
| Rates per 100 thousand person-year; ASR = Age standardized rate | | | | | |

| **Table s4. Hospitalization rates for migraine (ICD-10: G43)** | | | | | |
| --- | --- | --- | --- | --- | --- |
| **Year** | **Cases** | **Crude** | **ASR** | **95% Confidence Interval** | |
| 2013 | 579 | 36.70 | 36.40 | 33.57 | 39.48 |
| 2014 | 566 | 35.31 | 34.98 | 32.32 | 37.87 |
| 2015 | 579 | 35.57 | 35.10 | 32.43 | 37.93 |
| 2016 | 625 | 37.81 | 37.22 | 34.29 | 40.48 |
| 2017 | 539 | 32.13 | 31.68 | 28.91 | 34.49 |
| Rates per 100 thousand person-year; ASR = Age standardized rate | | | | | |

| **Table s5. Hospitalization rates for hydrocephalus (ICD-10: G91)** | | | | | |
| --- | --- | --- | --- | --- | --- |
| **Year** | **Cases** | **Crude** | **ASR** | **95% Confidence Interval** | |
| 2013 | 835 | 52.93 | 53.61 | 49.93 | 57.51 |
| 2014 | 898 | 56.03 | 56.74 | 52.87 | 60.78 |
| 2015 | 776 | 47.67 | 48.21 | 44.49 | 51.95 |
| 2016 | 864 | 52.27 | 53.25 | 49.59 | 57.01 |
| 2017 | 795 | 47.39 | 47.86 | 44.22 | 51.45 |
| Rates per 100 thousand person-year; ASR = Age standardized rate | | | | | |
